# Efficacies of different ovarian hyperstimulation protocols in poor ovarian responders classified by the POSEIDON criteria

**DOI:** 10.18632/aging.103210

**Published:** 2020-05-29

**Authors:** Fei Li, Tian Ye, Huijuan Kong, Jing Li, Linli Hu, HaiXia Jin, Yingchun Su, Gang Li

**Affiliations:** 1 Center for Reproductive Medicine, The First Affiliated Hospital of Zhengzhou University, and Henan Province Key Laboratory of Reproduction and Genetics, Henan, People’s Republic of China; 2 Center for Reproductive Medicine, The First People’s Hospital of Shangqiu, Henan, People’s Republic of China

**Keywords:** GnRH agonist, GnRH antagonist, POSEIDON criteria, poor ovarian response, controlled ovarian stimulation

## Abstract

We retrospectively analyzed clinical data from 45,912 *in vitro* fertilization/intracytoplasmic sperm injection cycles in our reproductive medical center. We compared the clinical outcomes of three different ovarian hyperstimulation protocols in poor ovarian responders (classified by the POSEIDON criteria) to determine the most effective protocol for each POSEIDON group. In POSEIDON groups 1 and 3, the early-follicular-phase long-acting GnRH-agonist long (EFLL) protocol was associated with higher pregnancy rates per transfer and higher live birth rates than the mid-luteal-phase short-acting GnRH-agonist long (MLSL) and GnRH-antagonist protocols. We also examined the relationship between advanced age and reproductive outcomes, and observed a negative correlation between age and live birth rate for each protocol (EFLL: OR = 0.890, 95% CI: 0.870 - 0.911, P < 0.001; MLSL: OR = 0.907, 95% CI: 0.885 - 0.926, P < 0.001; GnRH-antagonist: OR = 0.891, 95% CI: 0.857 - 0.926, P < 0.001). In terms of clinical outcomes, EFLL was the most effective protocol for young poor ovarian responders. However, there were no differences in the implantation rates, clinical pregnancy rates, or live birth rates among the protocols in older patients. Age is thus the most important determinant of oocyte quality, embryo ploidy, and delivery rate.

## INTRODUCTION

Poor ovarian response (POR) to controlled ovarian stimulation is one of the main challenges in assisted reproductive technology (ART) treatments [1]. The mechanisms underlying POR in ART remain unclear, so there is no consensus on the management of poor responders [2, 3]. Moreover, a wide diversity of criteria have been used to classify POR patients, even among studies performed in the same centers, so it has been difficult to identify the best protocol for this group of women. In 2011, a consensus was reached on the criteria to be used to accurately identify poor responders (the Bologna criteria) [4]; however, these criteria fail to reflect the significantly variable profiles and biological characteristics of POR patients, and do not account for the effect of age on oocyte quality [5, 6]. Moreover, these criteria do not include any recommendations for clinical practice.

In 2016, the Patient Oriented Strategies Encompassing IndividualizeD Oocyte Number (POSEIDON) group proposed a new stratification method for ART patients with reduced ovarian reserves or unexpected inappropriate ovarian responses to exogenous gonadotropins [7, 8]. This new classification system introduced a more nuanced picture of the “low prognosis patient” in ART. This method is applied retrospectively, and divides patients into four groups based on their age, anti-Müllerian hormone (AMH) levels, antral follicle counts (AFCs) and numbers of oocytes retrieved during the initial stimulation cycle [8, 9]. To date, POSEIDON stratification has been well accepted by infertility specialists and reproductive endocrinologists worldwide [10].

The POSEIDON criteria seem to be useful for identifying and classifying patients with impaired ovarian reserves or PORs, and for providing optimal guidance for the diagnosis and management of these patients [11]. However, there is little evidence supporting the validity of the parameters used in the POSEIDON criteria or the outcome assessments for different subgroups [12]. Thus, a large study comparing the effects of different ovarian hyperstimulation protocols for POSEIDON-stratified patients is necessary.

Gonadotropin-releasing hormone agonist (GnRH-a) treatment is an important component of controlled ovarian stimulation protocols for many patients. Since its development, GnRH-a treatment has increased patients’ retrieved oocyte numbers and pregnancy rates and reduced the number of cycle cancelations [13]. It is worth emphasizing that the early-follicular-phase long-acting GnRH-a long (EFLL) protocol was initially applied in a Chinese *in vitro* fertilization (IVF) center. In recent years, it has become the mainstream protocol in most reproductive medicine centers in China, including our own, due to its enhancement of endometrial receptivity, the pelvic microenvironment, embryo implantation and clinical pregnancy rates and its reduction of the abortion rate in the normal patient population [14].

Despite these advantages, the GnRH-a protocol may lead to ovarian hyperstimulation syndrome or other side effects [15]. Another method, the gonadotropin-releasing hormone antagonist (GnRH-ant) protocol, has been widely used in IVF/intracytoplasmic sperm injection (ICSI) for more than 20 years. In contrast to the GnRH-a long protocol, the GnRH-ant protocol avoids excessive pituitary suppression and flare-up side effects, requires a shorter usage duration and lower total dosage of gonadotropin, and reduces the incidence of severe ovarian hyperstimulation syndrome [15, 16]. Some studies have suggested that the GnRH-ant protocol is more convenient for patients than the GnRH-a long protocol, because the treatment time is shorter and fewer injections are needed [17]. There is reportedly no difference in the live birth rates of the GnRH-a and GnRH-ant protocols [18], and several similar studies have demonstrated that the GnRH-a long and GnRH-ant protocols have comparable efficacies in terms of IVF/ICSI outcomes in POR patients [15, 19].

Assuming that the GnRH-a and GnRH-ant protocols have comparable clinical outcomes, the benefits of the GnRH-ant protocol would justify a shift toward using it rather than the standard GnRH-a long protocol. However, the effectiveness and reliability of the GnRH-ant protocol are still debated [20, 21]. In several recent trials and meta-analyses, lower pregnancy rates and higher cancelation rates were observed with the GnRH-ant protocol than with the GnRH-a long protocol [22] or the GnRH-a short protocol, especially in patients with < 4 oocytes retrieved in previous controlled ovarian stimulation cycles or in those with expected POR, raising concerns about the effectiveness of the GnRH-ant protocol in poor ovarian responders [4, 23]. A series of studies suggested that the adverse effect of the GnRH-ant on endometrial receptivity is the main reason for the lower pregnancy and clinical pregnancy rates with this protocol [24, 25], and this proposal warrants further investigation.

At our reproductive medical center, > 10,000 IVF/ICSI cycles are performed each year. In the present study, we retrospectively evaluated the efficacies of the EFLL, mid-luteal-phase short-acting GnRH-a long (MLSL) and GnRH-ant protocols in POR patients (classified according to the POSEIDON criteria) who had undergone IVF/ICSI treatments, with the goal of providing guidance for the diagnosis and management of such patients in future clinical practice.

## RESULTS

### Patient characteristics

**Figure 1:**
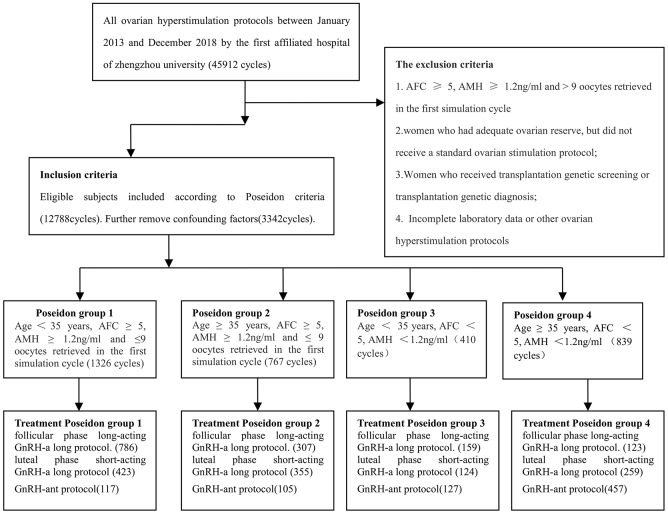
**Flowchart of patient recruitment between January 2013 and December 2018 at the First Affiliated Hospital of Zhengzhou University (45,912 cycles).**

We collected experimental data from the ovarian hyperstimulation protocols of 3,342 effective cycles between January 2013 and December 2018 at the First Affiliated Hospital of Zhengzhou University, after removing confounding factors and screening eligible subjects from a total of 45,912 cycles according to the POSEIDON criteria (Figure 1). We further categorized the eligible subjects into four groups (POSEIDON groups 1-4), and compared the baseline characteristics among patients who underwent the three different ovarian hyperstimulation protocols (EFLL, GnRH-ant and MLSL) in each POSEIDON group. A flow chart and the data processing procedure are shown in Figure 1. There were no significant differences in baseline characteristics such age, body mass index, basal follicle-stimulating hormone (FSH), basal luteinizing hormone (LH), basal estradiol and AMH levels among patients who underwent the three protocols in any of the four POSEIDON groups (Tables 1 and 2).

**Table 1:** Comparison of baseline parameters and clinical outcomes between the EFLL, MLSL and GnRH-ant protocols for embryo transfer cycles in POSEIDON group 1 and POSEIDON group 2.

**Group**	**POSEIDON group 1**				**POSEIDON group 2**			
**Protocols**	**EFLL**	**MLSL**	**GnRH-ant**	**P**	**EFLL**	**MLSL**	**GnRH-ant**	**P**
Age (years)	29.2±3.1	29.5±3.2	29.8±2.9	0.069	38.8±2.5	39.1±2.8	39.4±2.6	0.166
BMI (kg/m2)	22.6±3.3	22.5±3.2	22.3±3.2	0.679	23.2±2.9	23.2±3.4	22.8±2.9	0.455
Basal FSH (IU/L)	6.8±1.9	7.0±1.8	7.2±2.7	0.053	7.4±2.3	7.4±2.4	7.5±3.2	0.890
Basal LH (IU/L)	5.3±3.3	5.0±2.9	4.9±2.2	0.126	4.6±1.9	4.5±2.4	4.9±2.4	0.349
Basal E2 (ng/L)	43.8±101.2	40.2±23.2	45.8±40.0	0.691	41.4±35.3	42.4±29.9	41.5±29.7	0.915
Basal P (μg/L)	0.59±0.5	0.56±0.7	0.54±0.3	0.399	0.51±0.4	0.48±0.4	0.57±0.36	0.115
AMH (ng/mL)	3.4±2.2	3.3±2.6	3.7±3.3	0.159	2.7±1.5	2.8±1.6	2.7±2.4	0.607
Total dosage of Gn used (IU)	2633.5±934.6	2585.6±866.5	2737.2±856.1	0.303	3489.4±863.9	3065.5±729.3a	2973.9±722.9a	<0.001
Duration of Gn used (days)	13.5±2.1	11.3±1.9a	11.2±2.7a	<0.001	13.4±2.1	11.1±1.9a	10.3±2.4ab	<0.001
Oocyte number	12.8±6.4	10.8±5.8a	8.7±7.1ab	<0.001	7.7±3.52	8.1±5.0	4.9±3.2ab	<0.001
MII number	10.2±5.7	8.7±5.2a	6.8±5.9ab	<0.001	6.3±3.2	6.5±4.5	3.9±2.6ab	<0.001
Oocyte maturation rates (%)	79.4±18.5	80.2±19.8	76.8±25.6	0.232	82.0±18.3	79.6±25.7	80.3±24.7	0.260
Transferable embryos	4.3±2.8	2.8±2.4a	3.0±3.1a	<0.001	2.8±2	2.4±2.0a	2.1±1.7a	0.002
Good-quality embryos	3.7±2.6	3.0±2.9a	2.6±2.7a	<0.001	2.7±2.2	2.6±2.6	1.9±1.8ab	<0.001
ET cancellation (%)	22.6(178/786)	40.4(171/423)a	41.9(49/117)a	<0.001	19.5(60/307)	28.7(102/355)a	53.3(56/105)ab	<0.001
Fertilization rates (%)	60.3±22.3	60.2±24.3	57.5±28.9	0.339	62.3±25.3	62.1±25.3	61.5±30.8	0.959
Implantation rates (%)	42.8(481/1125)	40.3(199/493)	37.9(49/129)	0.427	19.8(91/460)	17.1(81/474)	28.7(26/94)	0.056
Pregnancy rates per transfer (%)	46.2(363/786)	34.5(146/423)a	31.6(37/117)a	<0.001	19.9(61/307)	18.3(65/355)	21.9(23/105)	0.693
Miscarriage rates (%)	13.5(49/363)	14.4(21/146)	13.5(5/37)	0.965	31.1(19/61)	32.3(21/65)	45.8(10/23)	0.543
Live birth rates per transfer (%)	39.7(312/786)	30.7(130/423)a	26.5(31/117)a	0.001	13.4(41/307)	12.4(44/355)	12.4(13/105)	0.926

Data are shown as means ± standard deviation or frequencies (percentages). BMI, body mass index; FSH, follicular-stimulating hormone; LH, luteinizing hormone; E2, estradiol; P, progesterone; AMH, anti-Müllerian hormone; ^a^P < 0.05, vs. early-follicular phase long-acting GnRH agonist long protocol (EFLL); ^b^P < 0.05, vs. Mid-luteal phase short-acting GnRH agonist long protocol (MLSL).

**Table 2:** Comparison of baseline parameters and clinical outcomes between the EFLL, MLSL and GnRH-ant protocols for embryo transfer cycles in POSEIDON group 3 and POSEIDON group 4.

**Group**	**POSEIDON group 3**				**POSEIDON group 4**			
**Protocols**	**EFLL**	**MLSL**	**GnRH-ant**	**P**	**EFLL**	**MLSL**	**GnRH-ant**	**P**
Age (years)	30.0±2.7	30.3±2.9	30.7±2.7	0.108	40.5±2.7	40.7±3.0	40.6±3.1	0.863
BMI (kg/m2)	22.2±3.2	22.7±3.4	22.8±3.2	0.270	23.7±3.0	23.7±2.6	23.3±2.6	0.066
Basal FSH (IU/L)	9.6±4.9	10.7±5.3	10.2±5.7	0.253	9.9±4.2	10.1±4.4	10.3±4.4	0.602
Basal LH (IU/L)	4.5±2.2	4.8±2.3	4.4±2.2	0.326	4.7±2.6	4.8±2.0	4.7±2.0	0.969
Basal E2 (ng/L)	46.1±32.0	44.9±36.5	43.4±30.7	0.794	47.0±28.8	48.9±40.2	46.6±32.6	0.678
Basal P (μg/L)	0.57±0.5	0.49±0.3	0.58±0.6	0.305	0.56±0.8	0.46±0.4	0.49±0.3	0.123
AMH (ng/mL)	0.57±0.2	0.55±0.3	0.51±0.3	0.145	0.54±0.2	0.54±0.3	0.53±0.3	0.959
Total dosage of Gn used (IU)	4084.1±786.2	3498.4±916.8a	3002.9±884.0ab	<0.001	4330.3±836.6	3707.7±904.8a	2812.6±718.9ab	<0.001
Duration of Gn used (days)	14.0±2.5	11.9±3.2a	10.2±2.8ab	<0.001	14.6±2.5	12.5±3.0a	9.4±2.4ab	<0.001
Oocyte number	5.6±3.6	4.6±3.2a	2.4±1.7ab	<0.001	3.9±2.8	3.1±2.3a	2.5±1.8ab	<0.001
MII number	4.6±3.0	3.7±2.8a	1.9±1.5ab	<0.001	3.1±2.2	2.5±2.0a	2.1±1.5ab	<0.001
Oocyte maturation rates (%)	84.1±23.8	78.3±28.2	76.5±34.8a	0.049	82.2±25.1	78.8±32.2	80.3±31.9	0.459
Transferable embryos	2.2±1.6	1.6±1.6a	1.1±1.1ab	<0.001	1.3±1.4	1.0±1.2a	1.2±1.1	0.026
Good-quality embryos	2.0±1.7	1.6±1.7a	1.0±1.1ab	<0.001	1.3±1.5	1.0±1.3	1.0±1.2	0.061
ET cancellation (%)	20.8(33/159)	39.5(49/124)a	69.3(88/127)ab	<0.001	40.7(50/123)	50.2(130/259)	66.7(305/457)ab	<0.001
Fertilization rates (%)	63.4±27.5	56.8±30.5	61.0±39.9	0.244	60.3±32.2	55.6±37.4	60.5±38.3	0.162
Implantation rates (%)	38.8(88/227)	32.8(43/131)	37.3(25/67)	0.529	18.1(21/116)	16.4(30/183)	20.3(52/255)	0.613
Pregnancy rates per transfer (%)	40.9(65/159)	29.0(36/124)a	15.7(20/127)ab	<0.001	13.0(16/123)	10.4(27/259)	10.1(46/457)	0.638
Miscarriage rates (%)	21.5(14/65)	19.4(7/36)	20.0(4/20)	0.764	25.0(4/16)	29.6(8/27)	30.4(14/46)	0.917
Live birth rates per transfer (%)	31.4(50/159)	23.4(29/124)	12.6(16/127)ab	<0.001	9.75(12/123)	7.34(19/259)	7.00(32/457)	0.584

Data are shown as means ± standard deviation or frequencies (percentages). BMI, body mass index; FSH, follicular-stimulating hormone; LH, luteinizing hormone; E2, estradiol; P, progesterone; AMH, anti-Müllerian hormone; ^a^P < 0.05, vs. early-follicular phase long-acting GnRH agonist long protocol (EFLL); ^b^P < 0.05, vs. Mid-luteal phase short-acting GnRH agonist long protocol (MLSL).

### Comparison of the three ovarian hyperstimulation protocols in different POSEIDON groups

We then compared the efficacies of the EFLL, MLSL and GnRH-ant protocols in each POSEIDON group. In POSEIDON group 1, the EFLL protocol was associated with a higher oocyte number (12.8 ± 6.4 vs. 10.8 ± 5.8 [MLSL] vs. 8.7 ± 7.1 [GnRH-ant], P < 0.001), higher number of metaphase II [MII] oocytes (10.2 ± 5.7 vs. 8.7 ± 5.2 vs. 6.8 ± 5.9, P < 0.001), longer duration of gonadotropin use (13.5 ± 2.1 d vs. 11.3 ± 1.9 d vs. 11.2 ± 2.7 d, P < 0.001), higher number of transferable embryos (4.3 ± 2.8 vs. 2.8 ± 2.4 vs. 3.0 ± 3.1, P < 0.001), higher number of good-quality embryos (3.7 ± 2.6 vs. 3.0 ± 2.9 vs. 2.6 ± 2.7, P < 0.001), lower embryo transfer [ET] cancelation rate (22.6% [178/786] vs. 40.4% [171/423] vs. 41.9% [49/117], P < 0.001), higher pregnancy rate per transfer (46.2% [363/786] vs. 34.5% [146/423] vs. 31.6% [37/117], P < 0.001) and higher live birth rate (39.7% [312/786] vs. 30.7% [130/423] vs. 26.5% [31/117], P = 0.001) than the MLSL and GnRH-ant protocols, respectively. There were no differences in the implantation rates, total gonadotropin dosages or miscarriage rates among patients who underwent the three ovarian hyperstimulation protocols in POSEIDON group 1 (Table 1).

In POSEIDON group 2, the EFLL protocol was associated with a higher total gonadotropin dosage (3489.4 ± 863.9 IU vs. 3065.5 ± 729.3 IU [MLSL] vs. 2973.9 ± 722.9 IU [GnRH-ant], P < 0.001), longer duration of gonadotropin use (13.4 ± 2.1 d vs. 11.1 ± 1.9 d vs. 10.3 ± 2.4 d, P < 0.001), higher number of transferable embryos (2.8 ± 2 vs. 2.4 ± 2.0 vs. 2.1 ± 1.7, P < 0.001) and lower ET cancelation rate (19.5% [60/307] vs. 28.7% [102/355] vs. 53.3% [56/105], P < 0.001) than the MLSL and GnRH-ant protocols, respectively. Furthermore, the EFLL protocol was associated with a higher oocyte number (8.1 ± 5.0 vs. 4.9 ± 3.2, P < 0.001), higher number of MII oocytes (6.5 ± 4.5 vs. 3.9 ± 2.6, P < 0.001) and higher number of good-quality embryos (2.6 ± 2.6 vs. 1.9 ± 1.8, P < 0.001) than the GnRH-ant protocol, although these parameters did not differ between the EFLL and MLSL protocols (P ≥ 0.05). No differences in the implantation rates, pregnancy rates per transfer, miscarriage rates or live birth rates were observed among patients who underwent the three ovarian hyperstimulation protocols in POSEIDON group 2 (Table 1).

Table 2 displays the comparison of the EFLL protocol to the MLSL and GnRH-ant protocols in POSEIDON groups 3 and 4. In POSEIDON group 3, the EFLL protocol was associated with a higher oocyte number (5.6 ± 3.6 vs. 4.6 ± 3.2 [MLSL] vs. 2.4 ± 1.7 [GnRH-ant], P < 0.001), higher number of MII oocytes (4.6 ± 3.0 vs. 3.7 ± 2.8 vs. 1.9 ± 1.5, P < 0.001), higher total gonadotropin dosage (4084.1 ± 786.2 IU vs. 3498.4 ± 916.8 IU vs. 3002.9 ± 884.0 IU, P < 0.001), longer duration of gonadotropin use (14.0 ± 2.5 d vs. 11.9 ± 3.2 d vs. 10.2 ± 2.8 d, P < 0.001), higher number of transferable embryos (2.2 ± 1.6 vs. 1.6 ± 1.6 vs. 1.1 ± 1.1, P < 0.001), higher number of good-quality embryos (2.0 ± 1.7 vs. 1.6 ± 1.7 vs. 1.0 ± 1.1, P < 0.001), lower ET cancelation rate (20.8% [33/159] vs. 39.5% [49/124] vs. 69.3% [88/127], P < 0.001), higher pregnancy rate per transfer (40.9% [65/159] vs. 29.0% [36/124] vs. 15.7% [20/127], P < 0.001) and higher live birth rate (31.4% [50/159] vs. 23.4% [29/124] vs. 12.6% [16/127], P = 0.001) than the MLSL and GnRH-ant protocols, respectively. No differences in the implantation or miscarriage rates were observed among patients who underwent the three ovarian hyperstimulation protocols in POSEIDON group 3 (Table 2).

In POSEIDON group 4, the EFLL protocol was associated with a higher oocyte number (3.9 ± 2.8 vs. 3.1 ± 2.3 [MLSL] vs. 2.5 ± 1.8 [GnRH-ant], P < 0.001), higher number of MII oocytes (3.1 ± 2.2 vs. 2.5 ± 2.0 vs. 2.1 ± 1.5, P < 0.001), longer duration of gonadotropin use (14.6 ± 2.5 d vs. 12.5 ± 3.0 d vs. 9.4 ± 2.4 d, P < 0.001), higher total gonadotropin dosage (4330.3 IU ± 836.6 vs. 3707.7 IU ± 904.8 vs. 2812.6 ± 718.9 IU, P < 0.001), higher number of transferable embryos (2.8 ± 2 vs. 2.4 ± 2.0 vs. 2.1 ± 1.7, P < 0.001) and lower ET cancelation rate (19.5% [60/307] vs. 28.7% [102/355] vs. 53.3% [56/105], P < 0.001) than the MLSL and GnRH-ant protocols, respectively. Additionally, the EFLL protocol was associated with a higher number of good-quality embryos (2.6 ± 2.6 vs . 1.9 ± 1.8, P < 0.001) than the GnRH-ant protocol, although this parameter did not differ between the EFLL and MLSL protocols (P ≥ 0.05). Moreover, there were no differences in the implantation rates, numbers of good-quality embryos, pregnancy rates per transfer, miscarriage rates or live birth rates among patients who underwent the three ovarian hyperstimulation protocols in POSEIDON group 4 (Table 2).

### Influence of age on the live birth rate in POR patients

**Figure 2:**
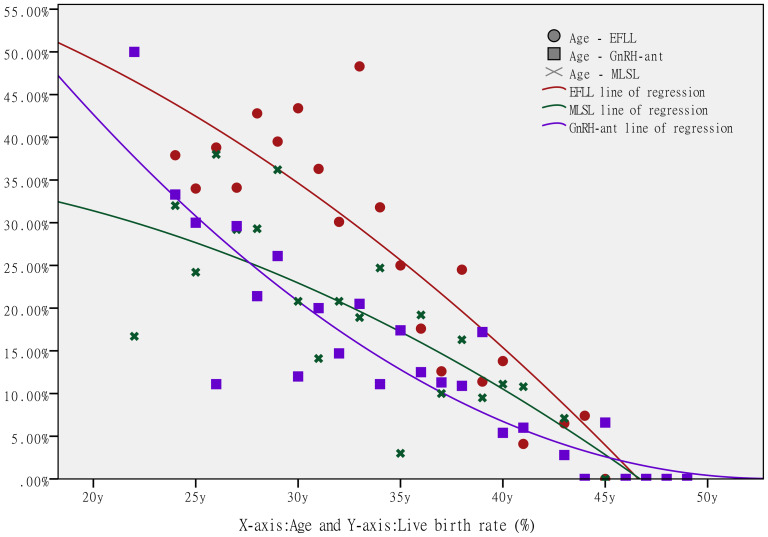
**The scatter plots and logistic regression lines of the live birth rate for each age group in the three protocol groups.** The circles (○ plot) represent the live birth rate for each age group in the EFLL protocol, the stars (× plot) represent the live birth rate for each age group in the MLSL protocol, and the boxes (□ plot) represent the live birth rate for each age group in the GnRH-ant protocol. There are three logistic regression lines, each representing the relationship between age and the live birth rate (red line: EFLL protocol, green line: MLSL protocol, purple line: GnRH-ant protocol). The x-axis represents age and the y-axis represents the live birth rate.

Age, which is a major criterion in the POSEIDON stratification, may be the most important determinant of oocyte quality and embryo ploidy. Because the live birth rate is the main indicator of ART success, further analysis of the relationship between age and this clinical outcome in POR patients is worthwhile. As shown in Figure 2, we calculated the live birth rate for each age group for each of the three protocols, and performed a scatter plot analysis. The scatter plot and logical regression line revealed a negative correlation between age and the live birth rate. Table 3 displays the results of the univariate logistic regression analysis for age and the live birth rate. For each ovarian hyperstimulation protocol, the live birth rate was significantly lower in patients ≥ 35 years old than in patients < 35 years old (EFLL: odds ratio [OR] = 0.890, 95% confidence interval [CI]: 0.870 - 0.911, P < 0.001; MLSL: OR = 0.907, 95% CI: 0.885 - 0.926, P < 0.001; GnRH-ant: OR = 0.891, 95% CI: 0.857 - 0.926, P < 0.001).

**Table 3:** Logistic regression analysis for age and live birth rate different ovarian hyperstimulation protocols.

**Factors**	**live birth rate**		
	**Exp (B)**	**95% C.I**	**P**
Age of Early-follicular phase long-acting GnRH-a long protocol	0.890	0.870-0.911	<0.001
Age of mid-luteal phase short-acting GnRH-a long protocol	0.907	0.885-0.926	<0.001
Age of GnRH antagonist protocol	0.891	0.857-0.926	<0.001

## DISCUSSION

The resulting pregnancy and live birth rates of a single attempted IVF/ICSI cycle are considered the primary endpoints for low prognosis patients undergoing ART treatments [2]. Previously, the clinical management of POR patients was primarily based on small studies [1, 26]. Because the mechanisms underlying POR in ART remain unclear [27], it has been difficult to develop clinical management strategies based on the characteristics and prognoses of patients, and there is no consensus on the management of poor responders [28]. Recently, the POSEIDON group proposed a new stratification method that has become well accepted by infertility specialists and reproductive endocrinologists worldwide [11]. With the POSEIDON stratification, a clinician can quickly evaluate whether a patient should be classified as a POSEIDON patient and determine which POSEIDON group is most appropriate [7, 11]. In this study, we assessed which protocol was most effective and reasonable for POR patients by comparing the clinical outcomes of the EFLL, MLSL and GnRH-ant protocols among patients in each POSEIDON group, with the goal of providing guidance for their diagnosis and management in future clinical practice.

For patients in POSEIDON groups 1 and 3, the EFLL protocol was more effective than the MLSL and GnRH-ant protocols, as evidenced by the higher oocyte number, higher number of transferable embryos, lower ET cancelation rate, higher pregnancy rate and higher live birth rate. However, for patients in POSEIDON groups 2 and 4, the pregnancy and live birth rates did not differ significantly among the three protocols, even though the EFLL protocol yielded higher numbers of oocytes and transferable embryos than the MLSL and GnRH-ant protocols. Thus, we recommend the EFLL protocol for patients in POSEIDON groups 1 and 3 because of its better clinical outcomes in these patients.

The intent of the EFLL protocol was to enable Chinese clinicians to improve endometrial receptivity, the pelvic microenvironment, the embryo implantation rate and the clinical pregnancy rate while reducing the abortion rate in normal or POR patients [13]. This protocol is designed to suppress the pituitary gland for 28 days, and a standard full dose of the GnRH-a before ovarian stimulation in IVF-ET might improve the pregnancy and live birth rates per fresh ET, although the exact mechanism remains unclear. Some studies have suggested that a full-dose depot GnRH-a injection for pituitary suppression in the EFLL protocol can increase endometrial receptivity for embryo implantation in young women [13, 29]. A study in an animal model suggested that preimplantation embryonic development was significantly enhanced when the embryos were incubated with increasing concentrations of GnRH-a, and the expression of GnRH and its receptor were also observed in human preimplantation embryos [13, 30].

In agreement with our findings, an early-stage meta-analysis on pregnancy outcomes revealed that the GnRH-a protocol was more effective than the GnRH-ant protocol [15, 25]. However, we want to emphasize that nonrandomized trials are always associated with selection bias issues. Despite our attempts to remove confounding factors and screen eligible subjects according to the POSEIDON criteria, patients with good ovarian responses may have been more likely to be assigned to the EFLL or MLSL group. Expected poor responders, especially those undergoing their second or third IVF/ICSI cycles, are more likely to be treated with the GnRH-ant protocol. Even so, the results of the GnRH-ant protocol establish a crude baseline that can be compared with the results of different protocols. We will conduct randomized controlled trials to confirm these findings in the future.

Based on the similar clinical outcomes of the three protocols among women of advanced age, we think that it is appropriate to use the GnRH-ant protocol in patients aged ≥ 35 years. This is because the GnRH-ant protocol uses lower doses of exogenous gonadotropin, reduces the cost of controlled ovarian stimulation and avoids the very deep suppression of endogenous FSH and LH concentrations in the early follicular phase at the stage of follicular recruitment, thus potentially improving the egg harvest [15, 19]. However, if patients in POSEIDON groups 2 and 4 require more controlled ovarian stimulation cycles to achieve better cumulative live birth rates, it will be necessary to re-evaluate the cost-effectiveness of the GnRH-ant protocol.

Our results indicated that the ET cancelation rate was higher for the GnRH-ant protocol than for the EFLL and MLSL protocols. The reason for this is unclear [16, 17], but it is possible that patients with lower ovarian reserves (e.g., POR patients) are prone to an early LH rise pre-ovulation, that they need sufficient GnRH-ant levels from the beginning, and that their follicles biologically mature quickly and are prone to premature luteinization. Other factors, such as a thinner endometrium, very few transferable embryos and high progesterone levels, may also contribute to a higher cancelation rate for the GnRH-ant protocol than for the EFLL or MLSL protocol [17, 21]. In our routine clinical practice, if the number of transferable embryos is less than three, the ET cycle might be canceled due to concerns about endometrial receptivity. A large retrospective analysis of young patients (< 35 years of age) also demonstrated that the cancelation rate prior to ET was higher for the GnRH-ant protocol than for the GnRH-a protocol [31].

Some studies have suggested that there are lower implantation rates for the GnRH-ant protocol than for the GnRH-a protocol due to differences in endometrial receptivity [23, 32]. However, in our study, the implantation rates did not differ among the three ovarian hyperstimulation protocols, regardless of the POSEIDON group. Additionally, a study using microarray data revealed that the gene expression profiles of endometrial cells after GnRH-ant treatment were more similar to those in natural cycles than to those after GnRH-a treatment [15, 19]. Overall, reports on endometrial receptivity for the GnRH-ant and GnRH-a protocols have been inconsistent, so further analysis is required.

Age is a major factor in the POSEIDON stratification, and baseline factors such as FSH levels, AFCs and AMH levels change with increasing age [33, 34]. POSEIDON group 1 patients undoubtedly exhibited the highest live birth rate due to their younger age and normal ovarian reserves, while POSEIDON group 4 patients exhibited the lowest live birth rate due to their advanced age and diminished ovarian reserves. However, we were interested to know whether patients in POSEIDON group 2 (older women with normal ovarian reserves) or POSEIDON group 3 (younger women with diminished ovarian reserves) would achieve better pregnancy outcomes. As shown in Tables 1 and 2, although patients in POSEIDON group 2 had better ovarian reserves and higher numbers of transferable embryos than those in POSEIDON group 3, the live birth rate was lower in POSEIDON group 2 than in POSEIDON group 3. This finding indicates that age may be the most important contributor to oocyte quality and embryo ploidy, which directly influence pregnancy outcomes. Female reproductive aging is a process dominated by the gradual decline in oocyte quantity and quality, and the number of harvested oocytes and metaphase II oocytes and embryo quality are the best predictors of reproductive outcome in women. There is a progressive decline in the ovarian reserve with a decrease in both the quantity and quality of oocytes, and this may negatively affect the pregnancy outcome of IVF/ICSI. We also observed a negative correlation between age and the live birth rate for each protocol (Figure 2 and Table 3).

Some studies have suggested that the embryo euploidy rate decreases by 2.4% per year with increasing female age, and that the blastocyst euploidy rate drops from 60% before 35 years to 30% after 40 years [33]. In fact, there is broad agreement in the literature that age-related changes in oocyte quantity and quality begin at 35 years of age [34]. The age of 35 was chosen as the cutoff point in the POSEIDON stratification, and is indeed the age at which aneuploidy rates begin to rise and implantation, pregnancy and live birth rates begin to decline in many large ART data sets [33, 35]. On the other hand, in POSEIDON group 1 vs. group 2 and group 3 vs. group 4, AFCs and AMH levels are very important criteria that influence predicted pregnancy outcomes. A large amount of research has established AFCs and AMH levels as reliable and accurate ovarian reserve tests in predicting the ovarian response [12].

In conclusion, the EFLL protocol was more effective than the MLSL and GnRH-ant protocols in terms of the clinical outcomes of young POR patients (POSEIDON groups 1 and 3). However, there were no differences in clinical outcomes such as the implantation, clinical pregnancy and live birth rates among the different protocols for older patients (POSEIDON groups 2 and 4). Age may be the most important determinant of oocyte quality and embryo ploidy, which directly impact pregnancy outcomes; the older the patient is, the lower the delivery rate will be. It is worth emphasizing that the characteristics and prognoses of patients should be used to develop clinical management strategies, especially for POR patients, and these strategies warrant further investigation.

## MATERIALS AND METHODS

### Patient inclusion and classification

This retrospective case-control study evaluated the efficacies of the EFLL, MLSL and GnRH-ant protocols in ART cycles for patients with POR classified by the POSEIDON criteria. We analyzed clinical data from 45,912 cycles of IVF/ICSI in our reproductive medical center. The experimental materials in this study were from the Clinical Reproductive Medicine Management System/Electronic Medical Record Cohort Database of the Reproductive Medical Center of the First Affiliated Hospital of Zhengzhou University. The data included demographics, ART history, protocol used, embryonic outcomes, pregnancies per transfer, miscarriage rates and live birth rates from cycles performed between January 2013 and December 2018. This study was approved by the Ethics Committee of the Reproductive Medicine Center of the First Affiliated Hospital of Zhengzhou University, China. Informed consent was waived with approval from the ethics committee. All research was performed in accordance with relevant guidelines and regulations.

We screened eligible subjects and removed confounding factors (e.g., PGD/PGS, incomplete laboratory data, loss of patient follow-up or other ovarian hyperstimulation protocols) from 45,912 cycles in our center to ensure that there were no statistically significant differences in baseline data (e.g., age, body mass index, basal FSH and AMH levels) in each group. The eligible subjects were categorized into four groups based on the POSEIDON criteria: POSEIDON group 1: age < 35 years, AFC ≥ 5, AMH ≥ 1.2 ng/mL and ≤ 9 oocytes retrieved in the first stimulation cycle (1326 cycles); POSEIDON group 2: age ≥ 35 years, AFC ≥ 5, AMH ≥ 1.2 ng/mL and ≤ 9 oocytes retrieved in the first stimulation cycle (767 cycles); POSEIDON group 3: age < 35 years, AFC < 5, AMH < 1.2 ng/mL (410 cycles); and POSEIDON group 4: age ≥ 35 years, AFC < 5, AMH < 1.2 ng/mL (839 cycles). The results (oocyte number, transferable embryo number, fertilization rate, live birth rate) of the EFLL, MLSL and GnRH-ant protocols were compared in each POSEIDON group.

### EFLL protocol

For patients undergoing the EFLL protocol (a new protocol developed by Chinese clinicians), we administered 3.75 mg of a long-acting GnRH-a (Diphereline, Beaufort-Ipson, France) on days 2-4 of menstruation. Patients were monitored by ultrasound, and serum sex hormone levels were measured. The following criteria were used for pituitary downregulation: LH < 5 IU/L, FSH < 5 IU/L, estradiol < 30 μg/mL and progesterone < 1 ng/mL; no functional cysts; follicle sizes of 3-5 mm by ultrasound; and induced ovulation. The starting dose of gonadotropin (Puregon, Organon, The Netherlands) was determined on the basis of the patient’s AFC, age, body mass index and previous ovarian response to stimulation. The dosage was adjusted continually according to the patient’s response. The trigger was normally administered with 250 μg of recombinant human chorionic gonadotropin (hCG) (Livzon Pharmaceuticals, China) and 2000 IU of urinary hCG (Merck Schlano, Italy) when dominant follicles measuring > 16 mm in diameter accounted for 60% of all follicles, or when a follicle reached 20 mm in mean diameter. Oocyte retrieval under the guidance of transvaginal ultrasound was performed 37 hours after the trigger.

### MLSL protocol

For patients undergoing the MLSL protocol (the conventional long protocol), 0.1 mg/day of a short-acting GnRH-a (Decapeptyl, Ferring GmbH, Germany) was used from the mid-luteal phase. After seven days, patients were monitored to ensure that there was no functional cyst and that the hCG test was negative. Then, the medication was continued for three days, and the dose was reduced to 0.05 mg/day for four days. The pituitary downregulation standard and trigger injection were identical to those of the EFLL protocol.

### GnRH-ant protocol

For the GnRH-ant protocol, ovarian stimulation was started with 112.5-300 IU of recombinant FSH (Puregon, Organon, The Netherlands) on day 3 of the menstrual cycle. The recombinant FSH dosage was adjusted according to the ovarian response, follicle size was determined by ultrasound, and serum hormone levels were measured. A daily dose of 0.25 mg of a GnRH-ant (Cetrotide, Pierre Fabre, France) was initiated when the lead follicle reached a mean diameter of 14 mm or on the sixth day of recombinant FSH stimulation, and the dose was continued until the day of hCG administration (250 μg of recombinant hCG [Livzon Pharmaceuticals] combined with 2000 IU of urinary hCG [Merck Schlano]). The hCG was injected after two or three leading follicles (at least 16 mm in diameter) had been confirmed by ultrasound and appropriate serum hormone levels had been measured.

### Statistical analysis

Continuous variables are expressed as means ± standard deviations, and were compared using one-way analysis of variance and the Bonferroni post-hoc test. Categorical variables are expressed as frequencies (percentages), and were compared using the chi-square test or Fisher’s exact test. Univariable logistic regression analyses were used to analyze the association between age and the live birth rate for each ovarian hyperstimulation protocol, and the ORs and 95% CIs were calculated. All analyses were performed with the Statistical Package for the Social Sciences (Version 19.0; SPSS, Chicago, IL, USA). P < 0.05 was considered to indicate statistical significance.
